# Prevalence and Risk Factors for Hearing Loss in Chilean Shellfish Divers

**DOI:** 10.29024/aogh.2310

**Published:** 2018-08-31

**Authors:** Marie Astrid Garrido Campos, Benedikt Anselm Hindelang, Denise Siqueira de Carvalho, Ilse Urzúa Finke, Ronald Herrera, Katja Radon

**Affiliations:** 1 Center for International Health at the Institute for Occupational, Social and Environmental Medicine, University Hospital, LMU Munich, Ziemssenstr. 1, 80336 Munich, DE; 2 Universidade Federal do Paraná, Curitiba, BR; 3 Mutual de Seguridad de la Cámara Chilena de la Construcción, Santiago, CL

## Abstract

**Background:**

Diving within artisanal fishing is a profession carried out by many men in coastal communities of southern Chile. These shellfish divers use surface supplied air for breathing. Among potential health threats are occupational accidents, decompression sickness and barotrauma. Repeated middle and inner ear barotrauma and decompression sickness of the ear may result in hearing loss.

**Objective:**

To determine the prevalence of hearing loss and related risk factors in artisanal shellfish divers.

**Methods:**

A cross–sectional study including 125 male shellfish divers was carried out in a coastal village in southern Chile. Participants were interviewed using a standard Spanish questionnaire adapted for this population. Hearing loss was assessed through audiometry. Any hearing loss, sensorineural hearing loss and other types of hearing loss (conduction, unilateral and mixed) were used as the outcomes. Bivariate and multiple logistic regression models were carried out to identify risk factors for hearing loss.

**Findings:**

Median duration on the job was 25 years (range 1–52), 64% of divers had a low level of schooling and 52% reported not knowing how to use decompression tables. Most (86%) of the divers dove deeper than 30 meters exceeding the 20 meters permitted by law. The majority (80%) reported having experienced several episodes of type II decompression sickness during their working life. The prevalence of any type of hearing loss was 54.4%: 29.0% presented sensorineural hearing loss and 25.6% presented other types of hearing impairment. After adjustment for age and other potential risk factors, diving more than 25 years was the main predictor for all kinds of hearing loss under study.

**Conclusions:**

Hearing loss is frequent in artisanal shellfish divers and safety measures are limited. Although based on small numbers and lacking an unexposed comparison group, our results suggest the need for community-based interventions.

## Introduction

Fishing is one of the most important economic activities of men living in villages in the southern Chilean coast. Shellfish divers extract seafood using compressed air as respiratory support. In contrast to scuba divers, shellfish divers use surface supplied air, also called Hookah technique. With this technique, compressed air is produced on the boat and piped to an air hose through which the diver inhales. The technique allows for longer time under the surface than Scuba diving. The other advantage of Hookah technique, particularly for Chilean shellfish divers, is the low legal requirements for the use of this technique. At the same time, the technique is only permitted up to a diving depth 20 meters, a depth at which almost no shellfish are available.

There are various hazards associated with this professional activity. In addition to fatal occupational accidents, the hyperbaric environment, cold water, sea current, and poor lighting might damage divers’ health [1,2,3,4]. Furthermore, ear disorders, such as middle or inner ear barotraumas (acute or repetitive), are common among divers [5,6]. These disorders are often caused by a deficient pressure balance. Likewise, decompression sickness of the inner ear, caused by nitrogen bubbles that obstruct blood vessels supplying the inner ear, might cause hearing loss [7,8,9]. Furthermore, noise-induced hearing loss due to motor noise has been described mainly among Scuba divers [10,11,12]. Overall, only a few studies have investigated the association between diving and hearing loss, and the results have been inconclusive [10,11,12,13,14]. None of the studies were carried out among artisanal shellfish divers using Hookah technique.

Professional divers in Chile might be insured against occupational accidents and diseases. This social insurance offers surveillance programs to protect from noise-induced hearing loss [15]. However, the majority of artisanal fishermen work for low-income under precarious working conditions without social protection, sufficient safety equipment, adequate training opportunities in occupational safety and health, and face an absence of surveillance programs.

Therefore, the aim of this study was to determine the prevalence of risk factors for hearing loss in shellfish divers in a coastal village of southern Chile. Based on this, governmental intervention programs should be established in the future.

## Methods

### Study Design and Participants

The cross-sectional study was carried out between November 2012 and January 2013 among male artisanal shellfish divers from a coastal village in southern Chile. Based on estimations by the health authority of the local fishing station, approximately 400 artisanal shellfish divers work in the village; however, due to the precarious working conditions, official registries do not exist. Nevertheless, many of the divers are associated with one of the five fishermen unions in the village. The union leaders of the village were informed about the project, and they provided their membership lists, including 243 shellfish divers. From this list, 75 divers were excluded for three reasons: 1) currently not working as a diver, 2) working as a diver outside the village or 3) death. The remaining 168 divers were visited in their homes or in public places and invited to participate in the study. In order to increase the number of participants, 60 shellfish divers who were not on the lists were invited while the researchers and the fishing station paramedic visited those identified (convenience sample).

Prior to participation, divers were informed about the purpose and activities of the project, the voluntary nature of participation and the confidentiality of information. Data collection was done anonymously. The study was approved by the ethical committees of the University Hospital in Munich (LMU) and Instituto de Seguridad del Trabajo in Chile.

Overall, 131 of 228 divers agreed to participate (57.5%), of which six were excluded from the hearing evaluation for the following reasons: earwax plug in the ear canal (n = 2), recent middle ear barotrauma (n = 1), perforated eardrum (n = 1), acute respiratory infection (n = 1) and known diagnosis of sudden hearing loss (n = 1). Consequently, 125 shellfish divers were included in the study.

### Data Collection and Study Instruments

The questionnaire and audiometry screening were conducted in the first aid room of the local fishing station. This room was a quiet location for the screening, without traffic or noisy machinery.

All divers were interviewed by two of the principal authors (MAG; BH) using a questionnaire based on the Spanish version of the European Working Condition and Health Survey [16]. Questions about level of education and age were included. To evaluate alcoholism, the Escala Breve de Beber Anormal (EBBA) was applied [17]. In addition, questions asking about diving conditions were prepared based on previous surveys [18,19]. The final questionnaire was expert validated by local experts. Furthermore, the comprehensibility of the questionnaire for the target population was evaluated in a pilot test including thirteen divers.

To evaluate hearing, field screening was performed by a trained technician measuring the frequencies from 500 Hz to 8000 Hz by air conduction. If thresholds were altered, bone conduction was also assessed. The definition of hearing loss was based on the criteria established in the Protocolo de Exposición Ocupacional a Ruido PREXOR of the Chilean Health Ministry [15], considering as altered audiometry a loss of ≥25 dBHL. To avoid mistakes in classification, the Pure Tone Average (PTA) for thresholds 500 to 3000 Hz [20] was additionally evaluated for each audiogram. Before the audiometry, divers’ ear canals were evaluated by otoscopy.

### Variable Definition

Four outcome definitions were used:

- Any type of hearing loss (yes vs. no): prevalence of any type of altered audiogram as described above.
- Sensorineural hearing loss (yes vs. no): audiogram suggestive of bilateral sensorineural hearing loss with a simultaneous decrease in the threshold of air and bone conduction. For bivariate and multivariate analysis divers with sensorineural hearing loss also identified with other types of hearing loss (see below) were excluded.
- Other types of hearing loss (yes vs. no): audiogram altered suggestive of conduction, unilateral and mixed hearing loss, after assessment of bone way and without a curve suggestive of bilateral sensorineural hearing loss. Divers identified with sensorineural hearing loss were excluded from this analysis. In order to assess potential risk factors for hearing loss, the following variables were considered:
- Age was categorized into ≥40 years vs. <40 years.
- Level of education was differentiated between those with some form of elementary education and those with higher level of education.
- Active smoker: Yes, if the diver reported to smoke occasionally or daily.
- Alcohol consumption: Yes, if the diver reported to drink alcohol occasionally or daily.
- Alcoholism: Yes, if there were two positive responses on the EBBA scale [17].
- Diving years: The duration of work as a shellfish diver was dichotomized using the median (25 diving years) as cut-off.
- Number of working days per week: only holidays and irregular days, Monday to Friday; Monday to Friday and exceptionally Sunday and/or holidays; Monday to Saturday; Monday to Sunday.
- Weekly dives: The number of dives per week were divided using 7 dives per week as cut-off.
- Diving duration: Average duration of the dives was determined by using 1 hour as cut-off.
- Ability to read decompression tables (yes vs. no).
- Use of decompression tables (yes vs. no).
- Deep dives: Dives exceeding depths of 30 meters.
- Middle ear barotrauma: Yes, if divers reported to have suffered barotrauma of middle ear while diving.
- Symptoms of type II decompression sickness (yes vs. no): Yes, if unexpected fatigue, headache, numbness/tingling, vertigo, loss of balance, difficulty breathing/choking, muscular weakness/paralysis, loss of vision, loss of consciousness, inability to urinate, or confusion/disorientation/memory loss had occurred after a dive more than once in their working life.

### Statistical Analysis

Statistical analysis was carried out using EpiInfo Version 3.5.4 through descriptive analysis, bivariate analysis (Chi^2^ test) and logistic regression analysis assessing the association between potential predictors and hearing loss.

In order to assess potential selection bias by the two different sampling methods, divers obtained from the lists of the fishermen unions were compared to those forming part of the convenience sample using the Chi^2^ test.

The associations between potential predictors and hearing loss was tested for all types of hearing loss and stratified for sensorineural and other types of hearing loss. All variables with a pChi^2^ < 0.10 for any type of hearing loss were included in the final multiple logistic regression model mutually adjusting for all other variables in the model.

## Results

No statistically significant differences were found between divers of fishermen unions (random sample) and those forming part of the convenience sample (Table 1). Thirty-two of the 125 shellfish divers were younger than 40 years (25.6%), two of them younger than 20 years. Young divers had a higher level of education than older divers (pChi^2^ < 0.01). Only 34% of shellfish divers had completed elementary education, 28% did not finish elementary education, and two divers had no formal education. The median duration of work as a diver was 25 years (range 1–52 years).

**Table 1:** Descriptive characteristics of southern Chilean shellfish divers. Total absolute and relative frequencies as well as stratified for sampling frame.

Characteristics	Total N = 125	Divers on list of fishermen’s unions N = 98	Divers not belonging to a union N = 27	P_Chi_^2^

	n (%)	n (%)	n (%)	

**Age (years) ≥ 40**	93 (74.4)	75 (76.5)	18 (66.7)	0.29
**Secondary education**	45 (36.0)	34 (34.7)	11 (40.7)	0.56
**Active smoker**	54 (43.5)	43 (44.3)	11 (40.7)	0.74
**Alcohol consumption**	91 (72.8)	70 (71.4)	21 (77.8)	0.51
**Alcoholism**	60 (48.0)	46 (46.9)	14 (51.9)	0.65
**Diving years (median with range)**	25 (minimum: 1.0; maximum: 52.0)	25 (minimum: 1.0; maximum: 52.0)	25 (minimum: 1.0; maximum: 34.0)	(p_Mann-Whitney-U-test_) 0.21
**Dives per week**				0.95
<or equal to seven dives	84 (67.2)	66 (67.3)	18 (66.7)	
More than seven dives	41 (32.8)	32 (32.7)	9 (33.3)	
**Diving duration**				0.56
<or equal 60 minutes	43 (34.4)	35 (35.7)	8 (29.6)	
More than 60 minutes	82 (65.6)	63 (64.3)	19 (70.4)	
**Ability to read decompression tables**	60 (48.0)	47 (48.0)	13 (48.1)	0.99
**Use of decompression tables**				0.73
Occasionally	44 (35.2)	34 (34.7)	10 (37.0)	
Always	54 (43.2)	44 (44.9)	10 (37.0)	
**Deep dives**	98 (78.4)	79 (80.6)	19 (70.4)	0.25
**Middle ear barotrauma (N_Missing_ 3)**	28 (23.0)	25 (26.3)	3 (11.1)	(p_Fisher exact_) 0.08
**Decompression sickness^##^**	100 (80.0)	82 (83.7)	18 (66.7)	0.05
**Audiometry**				0.63
-Normal	57 (45.6)	43 (43.9)	14 (51.9)	
-Suggestive of sensorineural hearing loss	36 (28.8)	30 (30.6)	6 (22.2)	
-Others (Suggestive of conductive hearing loss, unilateral hearing loss and mixed hearing loss)	32 (25.6)	25 (25.5)	7 (25.9)	

^##^Symptoms of type II decompression sickness more than once.

The prevalence of any type of hearing loss among shellfish divers was 54.4%. After bilateral sensorineural hearing loss (28.8%), the most common alteration of audiometry was unilateral hearing loss (14.4%). Only a few divers suffered from conduction hearing loss (5.6%) or mixed hearing loss (5.6%).

In the bivariate analysis, risk factors for all types of hearing loss were age ≥40 years and having more than 25 diving years. With respect to sensorineural hearing loss, being an active smoker was inversely related. For other types of hearing loss, having a lower level of education and not being able to read decompression tables were risk factors (Table 2).

**Table 2:** Prevalence of hearing loss in southern Chilean shellfish divers by potential risk factors.

	Any type of hearing loss			Sensorineural hearing loss			Other types of hearing loss		

	n (%)	N_Missing_	p_Chi_^2^	n (%)	N_Missing_	p_Chi_^2^	n (%)	N_Missing_	p_Chi_^2^

**Age (years)**			<0.01			<0.01^#^			<0.01^#^
<40	5 (15.6)			2 (6.90)			3 (10.0)		
≥40	63 (76.7)			34 (53.1)			29 (49.2)		
**Secondary education**			0.02			0.06			0.04
No	50 (62.5)			26 (46.4)			24 (44.4)		
Yes	18 (40.0)			10 (27.0)			8 (22.9)		
**Active smokers**		1	0.02			0.01		1	0.26
No	44 (62.9)			26 (50.0)			18 (4.9)		
Yes	23 (42.6)			10 (24.4)			13 (29.5)		
**Regular alcohol consumption**			0.54			0.73			0.50
No	20 (58.8)			10 (41.7)			10 (41.7)		
Yes	48 (52.7)			26 (37.7)			22 (33.8)		
**Alcoholism**			0.34			0.20			0.81
No	38 (58.5)			22 (44.9)			16 (37.2)		
Yes	30 (50.0)			14 (31.8)			16 (34.8)		
**Diving years**			<0.01			<0.01			<0.01
≤25	22 (31.9)			9 (16.1)			13 (21.7)		
>25	46 (82.1)			27 (73.0)			19 (65.5)		
**Nº of dives per week**			0.52			0.24			0.87
≤7	44 (52.4)			21 (34.4)			23 (36.5)		
>7	24 (58.5)			15 (46.9)			9 (34.6)		
**Diving duration**			0.82			0.59			0.84
≤1 hour/dive	24 (55.8)			14 (42.2)			10 (34.5)		
>1 hour/dive	44 (53.7)			22 (36.7)			22 (36.7)		
**Deep dives more than 30 meters**			0.11			0.21			0.18
No	11 (40.7)			6 (27.3)			5 (23.8)		
Yes	57 (58.2)			30 (42.3)			27 (39.7)		
**Ability to read decompression tables**			0.04			0.31			0.02
No	41 (63.1)			19 (44.2)			22 (47.8)		
Yes	27 (45.0)			17 (34.0)			10 (23.3)		
**Use of decompression tables**			0.54			0.46			0.81
Never	16 (59.3)			9 (45.0)			7 (38.9)		
Occasionally	24 (54.5)			13 (39.4)			11 (35.5)		
Always	28 (51.9)			14 (35.0)			14 (35.0)		
**Middle ear barotrauma**		3	0.42		3	0.66		1	0.36
No	49 (52.1)			26 (36.6)			23 (33.8)		
Yes	17 (60.7)			8 (42.1)			9 (45.0)		
**Decompression sickness^##^**			0.11			0.06^#^			0.42
No	10 (40.0)			4 (21.1)			6 (28.6)		
Yes	58 (58.8)			32 (43.2)			26 (38.2)		

^#^: p_Fisher Exact_. ^##^Symptoms of type II decompression sickness more than once.

After mutually adjusting for the other predictors in the model, diving years were associated with any type of hearing loss (OR 5.4 CI 95% 2.0–14.7), sensorineural hearing loss (OR 9.2 CI 95% 2.6–32.6) and other types of hearing loss (OR 3.7 CI 95% 1.2–11.7). Age was associated with any type of hearing loss (OR 5.4 95% CI 1.5–19.1) and when stratifying, it was related to other types of hearing loss only (OR 6.1 95% CI 1.1–32.0. Table 3). Smoking tended to be inversely related to sensorineural hearing loss in the adjusted model (OR 0.3 CI 95% 0.1–1.0).

**Table 3:** Crude (cOR) and adjusted (aOR) odds ratio for different types of hearing loss among shellfish divers in southern Chile.

Variables	Any type of hearing loss		Sensorineural hearing loss		Other type of hearing loss	

	cOR 95% CI	aOR 95% CI	cOR 95% CI	aOR 95% CI	cOR 95% CI	aOR 95% CI

**Age (years) ≥ 40**	11.3 (4.0–32.4)	5.4 (1.5–19.1)	15.3 (3.4–69.8)	4.4 (0.8–25.1)	8.7 (2.4–31.8)	6.1 (1.1–32.0)
**<40**	1	1	1	1	1	1
**Secondary education: Yes**	0.40 (0.2–0.9)	0.9 (0.3–2.5)	0.43 (0.2–1.1)	0.9 (0.2–3.2)	0.4 (0.1–1.0)	0.75 (0.2–2.7)
**No**	1	1	1	1	1	1
**Active smoker**	0.44 (0.2–0.9)	0.5 (0.2–1.2)	0.32 (0.1–0.8)	0.34 (0.1–1.0)	0.61 (0.3–1.5)	0.64 (0.2–1.8)
**Non-smoker**	1	1	1	1	1	1
**Diving years > 25**	9.83 (4.2–23.0)	5.41 (2.0–14.7)	14.1 (5.1–39.0)	9.20 (2.6–32.6)	6.90 (2.6–18.3)	3.7 (1.2–11.7)
**Diving years ≤ 25**	1	1	1	1	1	1
**Ability to read decompression tables: Yes**	0.50 (0.2–1.0)	0.83 (0.3–2.2)	0.70 (0.3–1.5)	1.38 (0.4–4.6)	0.33 (0.1–0.8)	0.62 (0.2–2.0)
**No**	1	1	1	1	1	1
**Decompression sickness^#^: Yes**	2.1 (0.9–5.1)	0.71 (0.2–2.4)	2.9 (0.9–9.4)	0.68 (0.1–3.3)	1.55 (0.5–4.5)	0.53 (0.1–2.2)
**No**	1	1	1	1	1	1

^#^Symptoms of type II decompression sickness more than once.

## Discussion

This study indicates a high prevalence of hearing loss among professional shellfish divers in southern Chile. More than 50% of those with a hearing impairment had an audiometric curve suggestive of sensorineural hearing loss. The main predictor for sensorineural and other types of hearing loss were diving years while age was only associated with other types of hearing impairment.

A high prevalence of hearing loss has been described in some other studies among professional divers [21,22,23,24]. In two of them, sensorineural hearing loss had an overall prevalence among 71% and 80% of divers [21,22]. In contrast, no increased prevalence of hearing loss was seen among sport divers and recreational SCUBA divers [13,14].

As known from the literature [25,26] age was one of the main predictors of hearing loss. Interestingly, when stratifying for type of hearing loss, the association remained only statistically significant for other types of hearing loss but not for sensorineural hearing loss. For the latter, the duration of diving was the most important predictor–however, the confidence interval was large due to the relatively limited number of divers (OR 9.2 CI 95% 2.6–32.6). These results are in line with a recent Peruvian study where age and diving years were also the main predictors of sensorineural hearing loss [21]. These similarities suggest that long-term exposure to risk factors in the work environment of shellfish divers can have a great influence on sensorineural hearing loss. Whether this hearing loss might is a result of the noise in the diver’s working environment or the diving itself has been discussed in several studies [6,10,11,12]. In contrast to the generally assumed negative impact of smoking on hearing [27,28], we found an inverse relationship. A similar relationship was discovered in a prospective study among European sport divers [10]. One might argue that, at least in our study, smokers were younger and thus, the impact of age on hearing loss is reflected also in the association between smoking and hearing loss.

Recurring symptoms of type II decompression sickness were not associated with sensorineural hearing loss. Different symptoms of the disease were combined so that the association still might hold true for decompression sickness of the inner ear. Accordingly, future research should include an assessment focused on the history of inner ear decompression sickness and inner ear barotrauma in order to establish possible associations for cumulative damage.

Having a high level of education and the ability to read decompression tables were protective factors for other types of hearing loss in the bivariate analysis. It is plausible that these factors contribute to risk-adverse diving behavior and indicate that noise may not be the main risk factor for hearing loss in the group under evaluation. However, future studies including a larger number of divers are necessary in order to explore this question.

Limitations of this study include the absence of an official and updated list of shellfish divers and having a small study group with a response is not a rate as it does not involve time of 55%, which made it difficult to generalize the results to the entire population of shellfish divers. In addition, we could not include an unexposed comparison group. One may question if a selection bias exists: for example, divers who previously experienced a hearing impairment may have been more likely to participate in the study. However, less than half of the divers with altered audiometry reported feeling their hearing impairment, which allows us to assume that no such selection bias was present.

Due to poor access to a hearing evaluation center, the only way to assess hearing loss among shellfish divers was through field screening audiometry. These conditions were adequate for an audiometric screening, considering that the purpose of this study was not to calculate the percentage of hearing loss. Other factors not included in this study were possible noise exposure in motorboats and the presence of chronic diseases that can affect the inner ear [10,12,25,29].

In conclusion, this study indicates that long-term professional diving is a risk factor for hearing loss in artisanal shellfish divers. Whether this is due to noise exposure or the hyperbaric environment remains a question for future research. Surveillance programs and community-based interventions should be considered to evaluate occupational diseases and risk factors and to determine preventive measures to avoid disability among these workers, the majority of who are not covered by social insurance against accidents and occupational diseases.
